# Cryogenically Deposited Plasmonic Au Nanoparticle Films for Photothermal Treatment of Lung Cancer Cells

**DOI:** 10.1021/acsomega.6c06589

**Published:** 2026-10-27

**Authors:** Vagif Nevruzoglu, Murat Tomakin, Nazlıhan Aztopal, Melih Manir, Tugba Bayazıt, Fatma Kurul, Bilge Can, Arif E. Cetin

**Affiliations:** † Department of Energy Systems Engineering, 175650 Recep Tayyip Erdogan University, Rize 53100, Turkey; ‡ Department of Physics, Recep Tayyip Erdogan University, Rize 53100, Turkey; § Department of Molecular Biology, Art and Science Faculty, Recep Tayyip Erdogan University, Rize 53100, Turkey; ∥ Central Research Laboratory, Recep Tayyip Erdogan University, Rize 53100, Turkey; ⊥ 605730 Izmir Biomedicine and Genome Center, Balcova, Izmir 35340, Turkey; # Department of Electrical and Electronics Engineering, 553310 Istanbul Atlas University, Kagithane, Istanbul 34408, Turkey; ¶ Department of Biophysics, Faculty of Medicine, Dokuz Eylul University, Balcova, Izmir 35340, Turkey; ∇ Izmir International Biomedicine and Genome Institute, Dokuz Eylul University, Balcova, Izmir 35340, Turkey

## Abstract

In this study, Au nanoparticle-coated glass substrates were fabricated by a cryogenic nonequilibrium deposition method at substrate temperatures of 300 and 230 K for plasmonic photothermal therapy applications. Field-emission scanning electron microscopy analysis revealed that the Au films deposited at 300 and 230 K consisted of nanoparticle assemblies with size distributions of approximately 7–17 nm and 8–10 nm, respectively. The significantly narrower particle size distribution observed at 230 K could be attributed to the emergence of a morphology consistent with the proposed soliton-mediated nonequilibrium growth mechanism characteristic of cryogenic deposition conditions. Atomic force microscopy measurements demonstrated that the average surface roughness decreased from 5.27 nm for the 300 K sample to 1.55 nm for the 230 K sample, confirming the formation of a more homogeneous nanoparticle architecture under cryogenic growth conditions. Optical characterization showed that both Au nanoparticle films exhibited surface plasmon resonance near 600 nm. However, the cryogenically fabricated film produced at 230 K displayed a substantially stronger plasmonic response, which was attributed to its enhanced structural uniformity and improved interparticle plasmonic coupling. To evaluate their photothermal performance, human lung adenocarcinoma A549 cells and normal bronchial epithelial BEAS-2B cells were cultured on bare-glass and Au nanoparticle-coated substrates and subsequently exposed to ∼600 nm irradiation using a 450 mW light source. Morphological and fluorescence-based analyses revealed pronounced photothermal-induced cytotoxicity in A549 cells cultured on Au-coated surfaces, including reduced cell density, disruption of cell–cell interactions, membrane damage, and loss of cellular organization. In contrast, BEAS-2B cells largely preserved their morphology, adhesion behavior, and membrane integrity under identical treatment conditions. The strongest cytotoxic response was observed for the Au nanoparticle films fabricated at 230 K. These findings suggest that cryogenic nonequilibrium deposition can improve nanoparticle uniformity and plasmonic performance, with the 230 K Au nanoparticle films producing the most pronounced photothermal response under the investigated conditions. In the preliminary biological evaluation, A549 cells exhibited a greater photothermal response than BEAS-2B cells under identical experimental conditions. These proof-of-concept findings highlight the potential of cryogenically fabricated plasmonic nanostructures for further investigation in photothermal biomedical applications.

## Graphical abstract

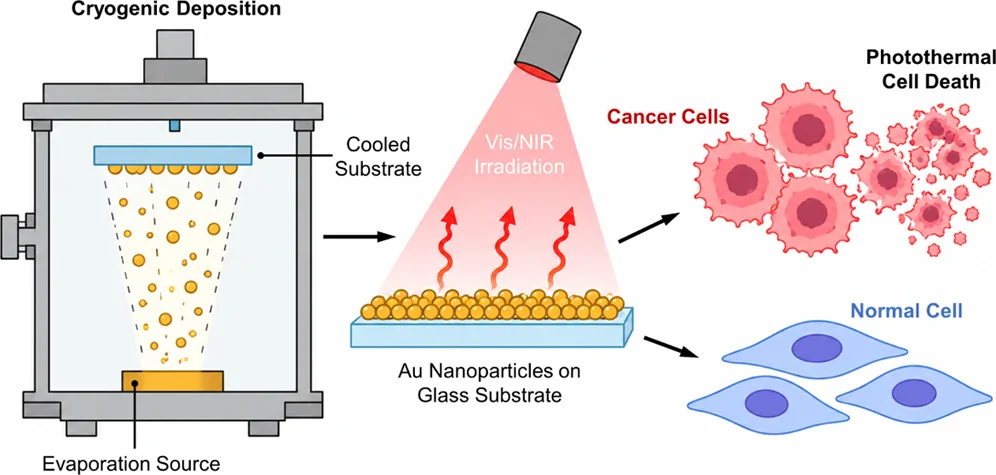

## 1 Introduction

Cancer remains one of the leading causes of mortality worldwide and continues to represent a major challenge for modern medicine because of its increasing incidence, high mortality rate, and limited therapeutic efficiency. According to recent global cancer statistics, approximately 19.2 million new cancer cases and nearly 9.9 million cancer-related deaths were reported in 2020, and the number of diagnosed patients is expected to increase dramatically over the next two decades.^1^ Among different cancer types, lung cancer is one of the most aggressive and deadly malignancies worldwide.^2,3^ Lung cancer can be classified into different histological types, with nonsmall cell lung carcinoma (NSCLC) and small cell lung carcinoma (SCLC) representing the two major categories. Nonsmall-cell lung cancer (NSCLC) accounts for nearly 80% of all lung-cancer cases, and adenocarcinoma is the most common histological subtype. Among them, NSCLC is responsible for nearly 85% of all lung-cancer cases, and adenocarcinoma is the most common histological subtype.^4,5^ Approximately 10–15% of patients exhibit activating mutations in the epidermal growth factor receptor (EGFR), which significantly influences disease progression and treatment response.^6^ Despite major advances in cancer biology and therapeutic technologies, the survival rate of lung-cancer patients remains critically low because most patients are diagnosed at advanced stages of the disease.^7^

Currently, surgery,^8^ chemotherapy,^9^ radiotherapy,^10^ nuclear medicine applications,^11^ and targeted therapies^12^ are among the primary treatment strategies used in lung-cancer therapy.^13^ Although surgical intervention remains highly effective for localized primary tumors, conventional therapeutic approaches still suffer from several critical limitations, including severe side effects, nonspecific toxicity toward healthy tissues, therapeutic resistance, and limited long-term efficiency.^14^ Chemotherapeutic agents generally circulate throughout the body and destroy rapidly proliferating cancer cells; however, the accumulation of toxic compounds within healthy tissues frequently leads to undesirable systemic side effects.^15^ In addition, cancer cells can progressively develop resistance against chemotherapeutic drugs, substantially reducing treatment efficiency. Similarly, radiotherapy relies on the application of high-energy particles such as X-rays, γ rays, or electrons to destroy cancerous tissues.^16^ Although radiotherapy is widely used as a complementary treatment following surgery, radiation-induced damage to healthy tissues remains a major clinical concern.^17^ Therefore, the development of alternative minimally invasive treatment strategies with enhanced selectivity and reduced systemic toxicity has become increasingly important.

Among emerging therapeutic approaches, plasmonic photothermal therapy (PTT) has attracted considerable attention as a minimally invasive nanotechnology-based cancer-treatment strategy.^20^ In photothermal therapy, plasmonic nanomaterials convert absorbed optical energy into localized heat, inducing selective thermal destruction of cancer cells while minimizing damage to surrounding healthy tissues.^18^ In this context, gold (Au) nanoparticles have been extensively investigated for photothermal cancer therapy because of their excellent biocompatibility, tunable optical properties, chemical stability, and strong surface plasmon resonance (SPR) behavior in the visible and near-infrared spectral regions.^19,23^ The localized SPR effect generated by Au nanoparticles can significantly increase local temperature within tumor regions under optical irradiation, resulting in effective photothermal ablation of cancer cells while preserving healthy surrounding tissues.^20^ Several previous studies have demonstrated that Au nanoparticle-mediated plasmonic heating can effectively induce selective cancer-cell destruction through localized hyperthermia processes.^21,22^ El-Sayed et al. demonstrated photothermal destruction of epithelial carcinoma cells using anti-EGFR-conjugated Au nanoparticles.^23^ On the other hand, Huang et al. investigated the temperature requirements for photothermal destruction using immunotargeted Au nanoparticles.^24^ Au-based plasmonic nanostructures, including hollow Au nanoshells and Au nanorods, have subsequently been investigated for localized photothermal ablation and improved light-to-heat conversion efficiency. These studies collectively demonstrate that the photothermal response of Au nanostructures is closely associated with their plasmonic properties and resulting localized heat generation under optical irradiation.^25–30^ The optical and photothermal properties of Au nanoparticles strongly depend on nanoparticle size, morphology, interparticle spacing, and structural uniformity. It is well established that the SPR process occurs at different wavelengths depending on nanoparticle geometry and nanoscale organization.^31^ Furthermore, effective photothermal therapy generally requires optical excitation within the biological transparency window, e.g., ∼600–900 nm, where light penetration through healthy tissues is maximized. Previous studies have emphasized that highly uniform metallic nanoparticle assemblies exhibit stronger plasmonic coupling and enhanced SPR behavior compared with heterogeneous nanoparticle systems.^32–34^ Therefore, precise control of nanoparticle size distribution and surface organization is critically important for achieving efficient plasmonic photothermal conversion.

Currently, Au nanoparticles are predominantly synthesized using chemical methods involving toxic reducing agents and stabilizing compounds.^35^ Such conventional synthesis techniques frequently produce heterogeneous nanoparticle structures consisting of clusters ranging from 1 to 100 nm in size. Previous reports have demonstrated that Au nanoparticles with sizes between approximately 15 and 30 nm exhibit highly effective photothermal therapeutic behavior.^36^ In contrast, ultrasmall Au nanoparticles (<3–5 nm) generally do not exhibit efficient SPR behavior and may accumulate within biological tissues, potentially causing undesirable toxicological effects.^39^ On the other hand, excessively large Au nanoparticles (50–100 nm) tend to aggregate within tumor regions, resulting in reduced therapeutic efficiency and nonuniform heat distribution during photothermal treatment.^37,38^ These findings demonstrate that the controllable fabrication of highly uniform Au nanoparticle assemblies is essential for improving plasmonic photothermal therapeutic performance.

Recently, cryogenic deposition approaches have emerged as promising strategies for the fabrication of highly uniform metallic and semiconductor nanostructures.^39^ The growth behavior of thin films prepared by physical vapor deposition is conventionally described using structure-zone models, in which film morphology is strongly influenced by the homologous substrate temperature (Ts/Tm) and the associated adatom mobility and diffusion behavior.^40^ Unlike conventional growth techniques, cryogenic deposition involves nanoparticle formation on cooled substrates under nonequilibrium conditions. Under such conditions, alternative growth pathways may contribute to nanoparticle nucleation and self-organization when conventional thermally activated surface diffusion is strongly suppressed. Under cryogenic growth conditions, film formation can proceed through soliton-mediated surface-wave dynamics, enabling the formation of densely packed and nearly monodisperse nanoparticle assemblies with enhanced structural homogeneity.^41^ Such highly ordered nanoparticle architectures are expected to significantly improve plasmonic coupling efficiency, localized electromagnetic field confinement, and photothermal energy conversion. Despite the significant progress achieved in plasmonic photothermal therapy, the fabrication of highly uniform Au nanoparticle assemblies with optimized plasmonic coupling and controlled photothermal performance remains a major challenge. In particular, the influence of cryogenic nonequilibrium growth processes and soliton-mediated nanoparticle self-organization on photothermal therapeutic efficiency has not yet been systematically investigated. Addressing this challenge requires the development of alternative nanofabrication strategies capable of simultaneously controlling nanoparticle size distribution, structural homogeneity, and plasmonic behavior.

Motivated by these considerations, this study explores a cryogenic nonequilibrium deposition approach for the fabrication of plasmonic Au nanoparticle films and investigates the relationship between nanoparticle growth regime, structural homogeneity, plasmonic characteristics, and photothermal therapeutic performance. FESEM analysis performed over a broad substrate-temperature range (100–300 K) revealed, for the first time, that a soliton-mediated growth regime emerges at a substrate temperature of 230 K, resulting in densely packed Au nanoparticle assemblies with an average particle size of approximately 12 nm and exceptional structural uniformity. Based on our previous theoretical studies, cryogenic nonequilibrium deposition has been proposed to promote a soliton-mediated self-organization process that may regulate nanoparticle nucleation and growth.^42,44,45^ The resulting plasmonic films were subsequently characterized in terms of their morphological, optical, and photothermal properties. To evaluate their biological performance, the fabricated Au nanoparticle films were tested using both human lung adenocarcinoma cells (A549) and normal bronchial epithelial cells (BEAS-2B) under visible-light irradiation conditions matched to the surface plasmon resonance wavelength of the Au nanostructures. The simultaneous evaluation of cancerous and noncancerous cell lines enabled assessment of the preferential response under the investigated experimental conditions for the proposed plasmonic platform and determination of whether cryogenically fabricated Au nanoparticle films could induce preferential photothermal damage toward cancer cells while minimizing nonspecific toxicity toward healthy epithelial cells. The obtained results demonstrate that cryogenic soliton-mediated growth can significantly enhance nanoparticle uniformity, plasmonic coupling, and photothermal cytotoxicity, highlighting the potential of this fabrication strategy for next-generation plasmonic photothermal therapy and other advanced biomedical applications.

## 2 Materials and Methods

Figure 1A schematically illustrates the cryogenic thermal evaporation system employed for the fabrication of plasmonic Au nanoparticle films.^42–44^ The coating system consisted of a liquid nitrogen-assisted cooling unit for controlling the substrate temperature and a quartz reactor chamber containing saturated Au vapor. High-purity metallic Au (99.999%, Sigma-Aldrich) was used as the evaporation source. During deposition, glass substrates were cryogenically cooled using liquid nitrogen and the substrate temperature was continuously monitored using a thermocouple. The deposition process was carried out under a high-vacuum environment of approximately 4.5 × 10^–6^ Torr. The structural, morphological, surface, and optical properties of the fabricated Au nanoparticle films were comprehensively characterized using multiple analytical techniques. Surface morphology was analyzed using a ZEISS GEMINI 500 field-emission scanning electron microscope (FESEM). Particle size distributions were obtained from representative FESEM micrographs using ImageJ software. Atomic force microscopy (AFM) measurements were carried out using a VEECO MULTIMODE 8 system to evaluate nanoscale surface roughness and topographical characteristics. The average roughness (*R*_a_) and root-mean-square roughness (*R*_q_) values were calculated to assess the effect of substrate temperature on nanoparticle uniformity and surface homogeneity. The optical transmittance (%T) and reflectance (%R) spectra of the Au nanoparticle films were measured at room temperature over the wavelength range of 400–800 nm using a SpectraMax M5 spectrophotometer and a Shimadzu reflectance measurement system, respectively.

**Figure 1:**
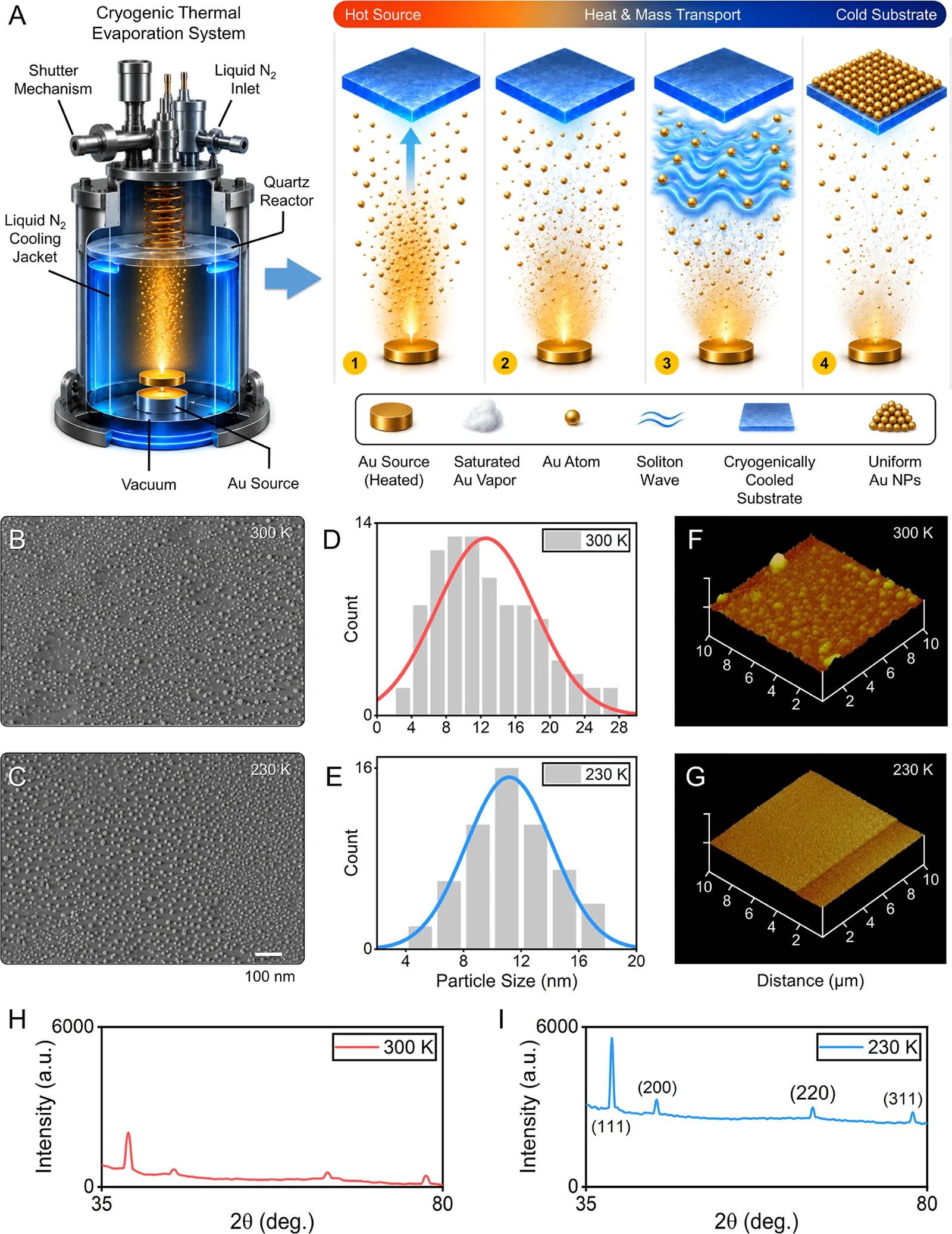
(A) Schematic illustration of the sequential stages involved in cryogenic nonequilibrium growth of plasmonic Au nanoparticle films. (B,C) FESEM images of Au nanoparticle films deposited at substrate temperatures of 300 and 230 K, respectively. (D,E) Particle size distribution histograms extracted from the FESEM images. (F,G) AFM surface topography images of Au nanoparticle films fabricated at 300 and 230 K, respectively. (H,I) XRD patterns of Au nanoparticle films deposited at substrate temperatures of 300 and 230 K, respectively.

To identify the substrate temperature at which the expected soliton-mediated growth mechanism emerges, Au films were fabricated under vacuum conditions over a substrate temperature range of 300–100 K. The fabrication mechanism proceeds through four sequential stages. In Step 1, the glass substrate is cryogenically cooled using liquid nitrogen while the Au source is simultaneously heated to thermally sublime the metallic Au, generating saturated Au vapor inside the reactor chamber. The substrate temperature was continuously monitored using a thermocouple system to maintain stable cryogenic nonequilibrium conditions during deposition. In Step 2, the generated Au vapor diffuses upward toward the cooled substrate surface. The shutter-controlled deposition geometry partially suppresses direct line-of-sight vapor flux, allowing a more homogeneous vapor transport regime within the chamber. In Step 3, interaction between the saturated Au vapor and the cryogenically cooled substrate is proposed to generate transient interfacial surface-wave dynamics that may facilitate soliton-mediated particle transport under cryogenic nonequilibrium conditions. These transient surface-wave dynamics are proposed to regulate the migration, nucleation, and self-organization behavior of Au atoms at the substrate interface. Cryogenic nonequilibrium deposition is therefore proposed to promote soliton-mediated self-organization of Au nanoparticles, resulting in highly uniform plasmonic films. Finally, in Step 4, according to the proposed soliton-mediated growth model, the associated particle-transport process may facilitate homogeneous nucleation and densely packed nanoparticle assembly, potentially resulting in Au nanoparticle films with a narrow size distribution and improved surface uniformity.

Based on the FESEM observations, Au/glass samples fabricated at 300 and 230 K were selected as representative structures exhibiting conventional island-type morphology and a highly uniform cryogenic morphology consistent with the proposed soliton-mediated growth model, respectively, and were subsequently used for optical characterization and photothermal therapy experiments. Human lung adenocarcinoma cells (A549) and normal bronchial epithelial cells (BEAS-2B) were used to evaluate the photothermal response of cancerous and noncancerous cells on Au nanoparticle-coated surfaces. Cells were cultured and maintained in 75 cm^2^ flasks containing RPMI 1640 medium or F-12K medium supplemented with 10% fetal bovine serum (FBS), penicillin, and streptomycin. Cell cultures were maintained in a humidified incubator at 37 °C under 5% CO_2_ atmosphere, and the culture medium was replaced every 2–3 days until ∼80% confluency was reached. Cells were subsequently detached using trypsin and collected for photothermal therapy experiments. Phosphate-buffered saline (PBS, pH 7.4) was used for washing procedures during cell preparation. Prior to photothermal treatment, A549 and BEAS-2B cells were seeded onto bare-glass and Au nanoparticle-coated glass substrates fabricated at 300 and 230 K at a density of 5 × 10^4^ cells per substrate and incubated for 8–10 h to allow cellular attachment and spreading. The experimental groups were designed to evaluate the effect of substrate morphology and visible-light illumination on cellular responses. Bare glass substrates were used as control surfaces, whereas Au nanoparticle-coated glass substrates fabricated at 300 and 230 K were employed as photothermal treatment surfaces. Human lung adenocarcinoma cells (A549) and normal bronchial epithelial cells (BEAS-2B) were cultured on each substrate type under illuminated and nonilluminated conditions. Two independent biological experiments were performed (*n* = 2), where PI-positive cells were quantified relative to the total number of DAPI-stained nuclei. Given the preliminary proof-of-concept nature of the biological experiments and the limited number of replicates, the quantitative biological data were evaluated descriptively rather than being used for inferential statistical comparisons.

## 3 Results

Figure 1B,C present the FESEM images of Au nanoparticle films fabricated on glass substrates at substrate temperatures of 300 and 230 K, respectively. As observed, the Au film deposited at 300 K consists of nanoparticle clusters with broad size distribution and nonuniform surface organization. In contrast, the Au film fabricated at 230 K exhibits densely packed nanoparticle assemblies with significantly improved size uniformity and homogeneous surface distribution. This morphology is consistent with the proposed soliton-mediated growth model under cryogenic nonequilibrium deposition conditions. FESEM analysis further showed that the morphological characteristics most consistent with the proposed soliton-mediated growth model were observed at a substrate temperature of 230 K, whereas the Au film deposited at 300 K exhibited conventional island-growth behavior typically observed in thermally driven deposition processes. Figure 1D,E show the particle size distribution analysis extracted from the FESEM images. The results show that the Au nanoparticle film fabricated at 230 K exhibits a substantially narrower particle size distribution compared with the film deposited at 300 K. In particular, the nanoparticle diameters of the cryogenically fabricated film were predominantly confined within a narrow range of approximately 11–13 nm, indicating highly controlled nucleation and growth behavior. Such enhanced structural uniformity is expected to promote more efficient and homogeneous surface plasmon resonance (SPR) characteristics, which are critically important for effective photothermal energy conversion during photothermal therapy applications.

Although the cryogenically fabricated Au nanoparticle films exhibited a substantially narrower particle size distribution and significantly improved surface homogeneity compared with films deposited at 300 K, these observations alone do not directly demonstrate a soliton-mediated growth mechanism. Alternative processes commonly associated with low-temperature deposition, including reduced surface diffusion, increased nucleation density, and kinetically limited island growth, may also contribute to the observed morphology. Nevertheless, the highly uniform nanoparticle organization obtained at 230 K is consistent with the previously proposed soliton-mediated nonequilibrium growth model, in which transient interfacial surface-wave dynamics regulate nanoparticle nucleation and self-organization during cryogenic deposition.^45–48^ Therefore, within the scope of the present study, the soliton-mediated growth mechanism should be regarded as a proposed interpretation that is consistent with the observed structural characteristics rather than as direct experimental proof. Further in situ experimental studies and theoretical modeling will be required to elucidate the dominant growth mechanism under cryogenic nonequilibrium deposition conditions. Figure 1F,G present the AFM surface topography images of the Au nanoparticle films fabricated at different substrate temperatures. The Au film deposited at 300 K exhibited an average surface roughness (*R*_a_) of 5.27 nm and a root-mean-square roughness (*R*_q_) of 6.61 nm. In contrast, the Au film fabricated at 230 K showed significantly lower roughness values, with an *R*_a_ of 1.55 nm and an *R*_q_ of 2.11 nm. The substantial reduction in surface roughness observed for the cryogenically fabricated film indicates improved surface homogeneity at 230 K and is consistent with the morphological characteristics expected under the proposed soliton-mediated growth model. Such enhanced morphological homogeneity is expected to contribute to more uniform plasmonic behavior and improved photothermal performance.

X-ray diffraction (XRD) analysis was performed using a Panalytical Empyrean diffractometer equipped with CuKα radiation (λ = 1.5408 Å) to investigate the crystal structure of the Au nanoparticle films. Figure 1H,I present the X-ray diffraction (XRD) patterns of the Au nanoparticle films deposited for 25 s at substrate temperatures of 300 and 230 K, respectively. Both samples exhibited the characteristic diffraction peaks of face-centered cubic (fcc) crystalline Au, corresponding to the (111), (200), (220), and (311) crystallographic planes (JCPDS No. 01-075-6560). The Au film fabricated at 230 K exhibited higher diffraction intensities than the film deposited at 300 K. However, diffraction intensity alone cannot be regarded as direct evidence of improved crystallinity, since it may also be influenced by differences in film thickness, the amount of deposited Au, and preferred crystallographic orientation. Therefore, the differences in peak intensity are interpreted cautiously as reflecting differences in the structural organization of the two Au films rather than as direct evidence of enhanced crystallinity. The structural parameters calculated from the XRD analysis are summarized in Table 1. The crystallite sizes estimated from XRD were 18.35 and 16.30 nm for the films deposited at 300 and 230 K, respectively. These values are somewhat larger than the nanoparticle dimensions determined from the corresponding FESEM images. This difference is not unexpected because XRD-derived crystallite size represents the dimension of coherently diffracting crystalline domains estimated from diffraction-peak broadening, whereas FESEM provides a direct morphological measurement of the lateral dimensions of individual surface nanoparticles or nanoparticle assemblies. Consequently, crystallite size obtained by XRD and particle size determined by FESEM do not necessarily correspond on a one-to-one basis. Nevertheless, both characterization approaches indicate clear substrate-temperature-dependent differences in the structural organization of the deposited Au films. Taken together with the FESEM and AFM observations, the XRD results provide complementary information on the crystalline structure of the Au films but should not be interpreted independently as evidence of improved crystallinity or as direct proof of the proposed soliton-mediated growth mechanism.

**Table 1:** Structural Parameters of Au Nanoparticle Films Deposited at Substrate Temperatures of 300 and 230 K, Calculated from the XRD Patterns

substrate temperature (K)	2θ (°)	*d* (Å)	*a* (Å)	*B* (°)	crystallite size, *D* (nm)	lattice strain, ε (×10^–2^)
300	38.7	2.35	4.04	0.46	18.35	1.89
230	38.4	2.36	4.08	0.54	16.30	2.13

The growth behavior of metallic thin films prepared by physical vapor deposition is commonly interpreted using the structure zone model (SZM), which describes film evolution as a diffusion-controlled process governed by the homologous temperature ratio, θ = *T*_s_/*T*_m_, where *T*_s_ is the substrate temperature and *T*_m_ is the melting temperature of the deposited material. Depending on this ratio, four characteristic growth regimes are generally distinguished. At low homologous temperatures (*T*_s_/*T*_m_ < 0.2; Zone 1), limited adatom mobility suppresses surface diffusion, resulting in porous or columnar microstructures with a high density of structural defects. As *T*_s_/*T*_m_ increases, enhanced surface diffusion promotes grain coalescence, improved crystallinity, and denser film formation.^49^ For Au films deposited at 230 K, the corresponding *T*_s_/*T*_m_ ratio places the growth process within Zone 1 of the SZM, where conventional theory predicts porous or columnar structures arising from kinetically limited surface diffusion. However, the FESEM images obtained in the present work reveal densely packed Au nanoparticle assemblies with a narrow particle size distribution rather than the irregular morphology typically associated with Zone 1 growth. AFM analysis further demonstrates substantially reduced surface roughness, while XRD confirms improved crystallinity and reduced structural disorder. These observations suggest that conventional diffusion-controlled growth alone may not fully explain the structural evolution of the cryogenically deposited films. Similar deviations from the predictions of the Structure Zone Model have previously been reported for cryogenically deposited metallic and plasmonic thin films, where enhanced structural quality was observed despite deposition within the low-temperature growth regime.^50,51^

To explain these observations, a soliton-mediated nonequilibrium growth model has previously been proposed. According to this model, nonlinear localized excitations generated at the film–substrate interface facilitate collective particle transport under cryogenic nonequilibrium conditions. Unlike conventional thermally activated surface diffusion, the proposed soliton-wave propagation enables coherent mass transport with minimal energy dissipation, allowing highly uniform nanoparticle self-organization even when thermal diffusion is strongly suppressed. In this model, particle migration preferentially occurs along Burgers-vector-related dislocation pathways at the substrate interface, thereby promoting directional self-organization and homogeneous nanoparticle assembly. Furthermore, only neutral clusters exceeding a critical size are expected to initiate soliton-wave propagation, providing a possible explanation for the narrow particle-size distributions observed experimentally.^52^ The empirical criterion proposed for soliton-wave formation is expressed as

$$\frac{a\left(T_{r}\right)-b\left(T_{f}\right)}{b\left(T_{f}\right)}>{\left(\frac{2}{\pi }\right)}^{3/2}\sqrt{\frac{f}{\lambda a\left(T_{r}\right)}}$$

where *a*(*T*_r_) and *b*(*T*_f_) represent the lattice parameters of the substrate and deposited material, respectively, while *f* and λ describe the interaction strength and wavelength associated with the nonlinear excitation. When this inequality is satisfied, the interface is considered favorable for soliton-wave propagation, enabling particle transport through a mechanism distinct from conventional thermally activated diffusion.^52^ Although the present FESEM, AFM, and XRD results are consistent with this previously proposed nonequilibrium growth model, they do not constitute direct experimental proof of soliton-mediated transport. Alternative mechanisms, including reduced surface diffusion, increased nucleation density, and kinetically limited island growth under cryogenic deposition conditions, may also contribute to the observed morphology. Therefore, within the scope of the present study, the soliton-mediated mechanism should be regarded as a proposed interpretation consistent with the experimental observations rather than as a directly verified growth mechanism. Future in situ experimental characterization together with theoretical modeling will be required to distinguish among these possible growth pathways.

Figure 2 presents the optical and plasmonic characterization of Au nanoparticle films fabricated under different growth regimes. As shown in the transmission spectra, both samples exhibit characteristic SPR behavior centered near 600 nm, confirming plasmon-assisted optical interaction within the visible spectral region. The observed SPR is noticeably red-shifted compared with the characteristic resonance of isolated spherical Au nanoparticles (typically ∼500 nm). This behavior is expected because the present samples consist of substrate-supported Au nanoparticle films rather than dispersed colloidal nanoparticles. In densely packed nanoparticle assemblies, reduced interparticle spacing promotes nearfield plasmonic coupling, leading to collective plasmon modes that shift the resonance toward longer wavelengths. In addition, the dielectric environment provided by the glass substrate, together with partial film continuity and variations in nanoparticle arrangement, may further contribute to the observed red shift. Therefore, the measured SPR band reflects the collective optical response of the nanoparticle film rather than the resonance of isolated Au nanoparticles.

**Figure 2:**
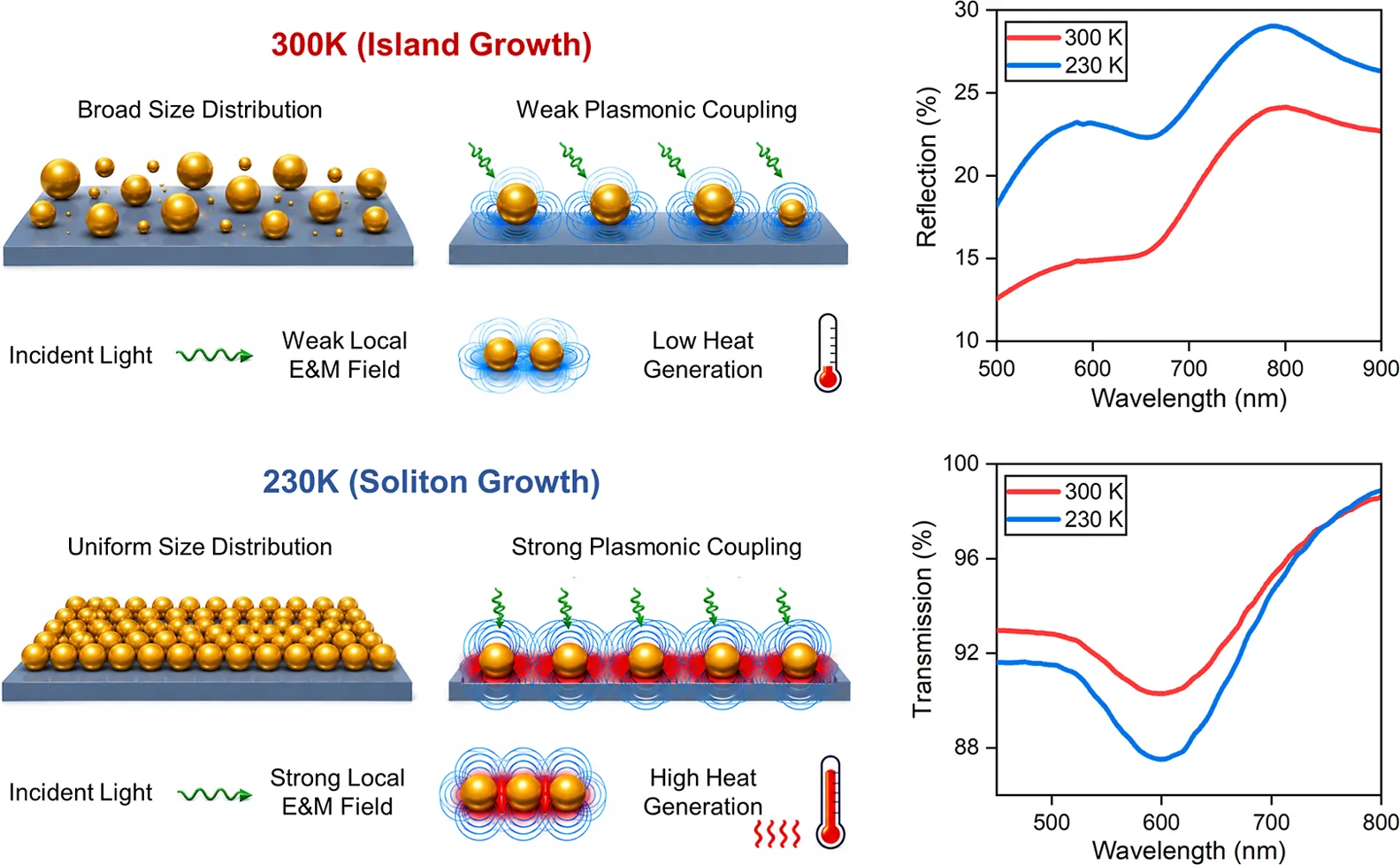
Schematic illustration comparing the plasmonic behavior and photothermal conversion mechanisms of Au films fabricated at 300 K (island-growth regime) and 230 K (soliton-mediated growth regime). Reflection and transmission spectra of Au nanoparticle films fabricated at 300 and 230 K. The cryogenically fabricated film exhibits enhanced plasmonic optical response associated with improved nanoparticle uniformity and densely packed nanoparticle assembly.

However, the Au film fabricated at 300 K displays a relatively weaker plasmonic response compared with the cryogenically fabricated film at 230 K. This weaker SPR behavior is attributed to the broad particle size distribution and nonuniform nanoparticle organization associated with the conventional island-growth mechanism. The presence of differently sized and randomly distributed Au nanoparticle clusters reduces collective plasmonic oscillation coherence and weakens near-field electromagnetic coupling between neighboring nanoparticles. In contrast, the Au film fabricated at 230 K exhibits significantly enhanced plasmonic behavior due to the highly uniform nanoparticle morphology generated through the proposed soliton-mediated nonequilibrium growth mechanism. The densely packed nanoparticle arrangement and narrow particle size distribution improve interparticle plasmonic coupling, resulting in stronger localized electromagnetic field confinement under visible-light excitation. As schematically illustrated in Figure 2, the cryogenically fabricated Au film promotes the formation of intense plasmonic hot spots under ∼600 nm light irradiation, which is expected to substantially enhance photothermal energy conversion efficiency.

The reflectance spectra further support the morphology-dependent optical behavior of the fabricated films. The Au film deposited at 230 K exhibits considerably higher reflectance values compared with the film fabricated at 300 K over the investigated spectral range. This enhanced reflectance behavior is attributed to the improved surface homogeneity and densely packed nanoparticle architecture observed in the FESEM and AFM analyses. The more uniform plasmonic surface minimizes random optical scattering losses while promoting coherent light–matter interaction across the Au nanoparticle film. The schematic comparison presented in Figure 2 summarizes the relationship between nanoparticle growth regime, plasmonic coupling behavior, and photothermal performance. The Au film fabricated at 300 K exhibits weak localized electromagnetic fields and limited heat generation due to random nanoparticle spacing and broad size dispersion. In contrast, the cryogenically fabricated film produced at 230 K generates stronger localized electromagnetic hot spots and enhanced photothermal heat generation owing to the highly ordered nanoparticle assembly and stronger plasmonic coupling behavior.

In order to investigate the relationship between nanoparticle growth regime, plasmonic behavior, and photothermal therapeutic efficiency, Au nanoparticle films fabricated under conventional and cryogenic nonequilibrium growth conditions were evaluated using in vitro photothermal therapy experiments. Cell morphology and density were initially evaluated before and after photothermal treatment using an EVOS M5000 Imaging System (Thermo Fisher Scientific). Following illumination, cell membrane integrity and photothermal-induced cell death were assessed using 4′,6-diamidino-2-phenylindole (DAPI) and propidium iodide (PI) staining. Detached cells were collected, washed with phosphate-buffered saline (PBS, pH 7.4; Sigma-Aldrich), and subsequently stained with DAPI and PI at a final concentration of 1 μg/mL for 30 min at room temperature in the dark. After staining, the cells were immediately imaged using the EVOS M5000 Imaging System. DAPI staining was used to visualize the total cell nuclei population, whereas PI-positive cells were considered membrane-compromised (nonviable) cells. Photothermal-induced cell death was quantified by calculating the percentage of PI-positive cells relative to the total number of DAPI-stained nuclei.

Since the photothermal performance of plasmonic nanostructures strongly depends on nanoparticle size distribution, surface homogeneity, and interparticle plasmonic coupling, the biological response of cells cultured on Au films fabricated at different substrate temperatures was systematically compared. Human lung adenocarcinoma cells (A549), one of the most extensively studied nonsmall-cell lung cancer (NSCLC) cell models, were selected as lung cancer remains among the leading causes of cancer-related mortality worldwide and represents an important target for emerging photothermal therapeutic strategies. In particular, the cryogenically fabricated Au films produced at 230 K were expected to exhibit enhanced photothermal activity due to their highly uniform nanoparticle organization and stronger localized electromagnetic field generation arising from the proposed soliton-mediated growth mechanism. Therefore, fluorescence-based membrane integrity analysis was performed to determine whether the enhanced plasmonic properties of the cryogenic Au nanoparticle films could translate into improved photothermal-induced cancer cell damage under visible-light irradiation.

Figure 3 presents the experimental workflow, plasmonic heating mechanism, fluorescence imaging results, and quantitative photothermal therapeutic response of A549 cells cultured on Au nanoparticle-coated glass substrates fabricated at different substrate temperatures. As schematically illustrated in Figure 3A, A549 were seeded onto Au nanoparticle-coated glass substrates fabricated at 230 and 300 K at a density of 5 × 10^4^ cells per substrate and incubated for 8–10 h at 37 °C under a humidified 5% CO_2_ atmosphere to allow cellular attachment and spreading. Following incubation, the samples were exposed to visible-light irradiation using a DongWoo Optron xenon light source operating at approximately 450 mW. The illumination wavelength range was specifically selected to overlap with the SPR region of the fabricated Au nanoparticle films. After photothermal illumination for 10 min, detached cells were collected, washed with PBS, and stained using DAPI and propidium iodide (PI) for fluorescence-based membrane integrity analysis. Fluorescence imaging was subsequently performed using an EVOS 5000 fluorescence microscope. The proposed plasmonic heating and cell-damage mechanism is schematically summarized in Figure 3B. Upon visible-light excitation, the Au nanoparticle-coated substrates absorb optical energy through localized surface plasmon resonance processes. In the cryogenically fabricated Au films, the highly uniform and densely packed nanoparticle arrangement promotes stronger plasmonic coupling and localized electromagnetic hot-spot generation. The resulting localized thermal confinement induces membrane disruption, increased membrane permeability, and enhanced cellular damage, allowing PI molecules to penetrate membrane-compromised cells. In contrast, Au films fabricated under conventional island-growth conditions exhibit weaker plasmonic coupling and reduced local heat generation due to their broader particle size distribution and nonuniform nanoparticle organization.

**Figure 3:**
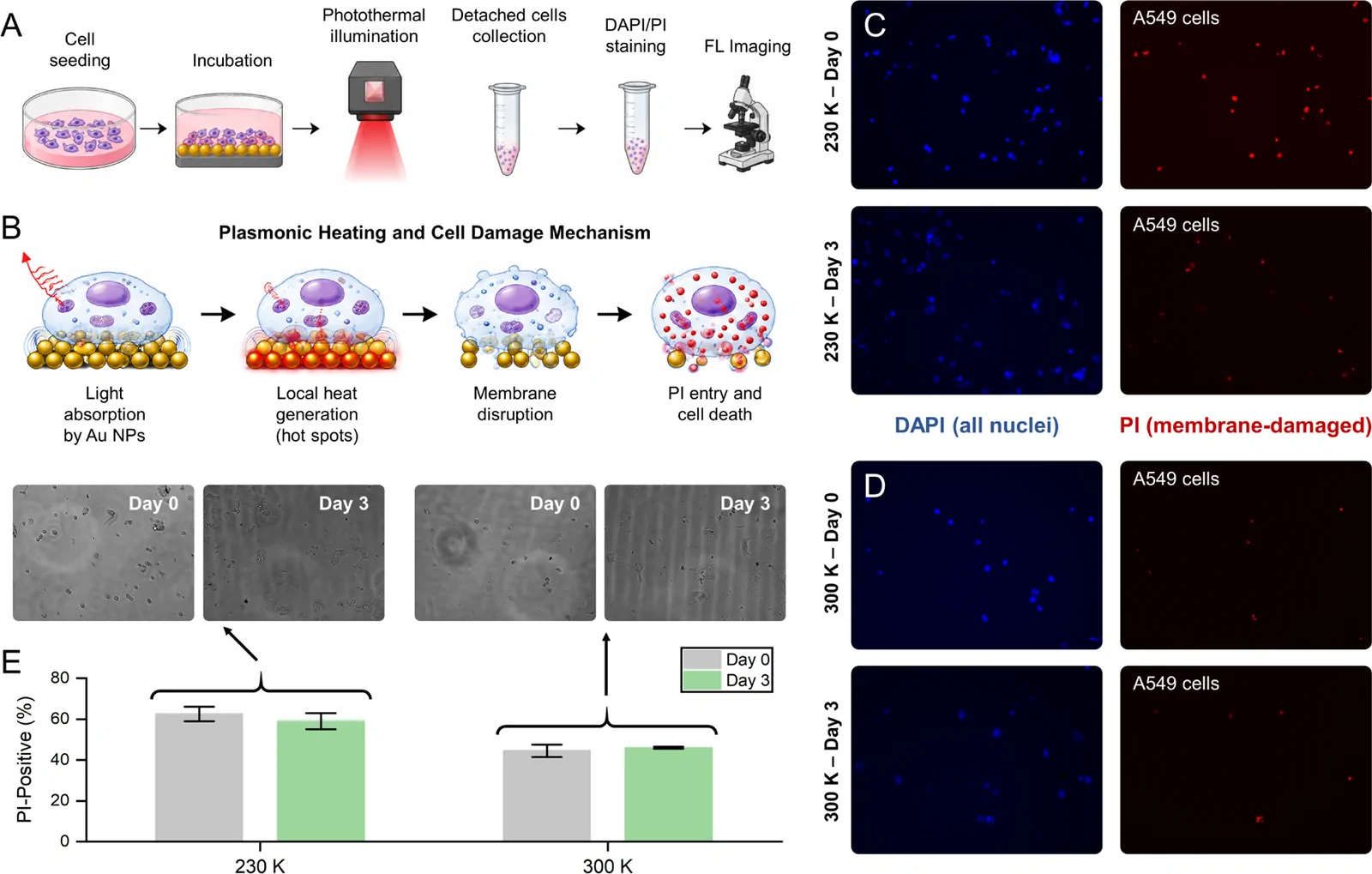
(A) Schematic illustration of the photothermal therapy experimental procedure. (B) Proposed plasmonic heating and membrane-damage mechanism induced by Au nanoparticle-mediated photothermal conversion under visible-light irradiation. (C,D) Representative DAPI (blue), and PI (red) fluorescence images of A549 cells cultured on Au nanoparticle-coated substrates fabricated at 230 and 300 K, respectively, following photothermal illumination. (E) Quantitative analysis of PI-positive cells normalized to total DAPI-stained nuclei. Data are presented descriptively as mean ± SD (*n* = 2), and no inferential statistical analysis was performed. Figure inset shows the brightfield images of A549 cells on Au nanoparticle-coated substrates.

Representative DAPI and PI fluorescence images of cells cultured on Au nanoparticle-coated substrates are presented in Figure 3C,D for 230 and 300 K samples, respectively. DAPI staining was used to visualize the total cell nuclei population, whereas PI-positive cells were considered membrane-compromised nonviable cells. The fluorescence images reveal substantially stronger PI-positive staining on the cryogenically fabricated Au surfaces compared with the conventionally fabricated samples. In particular, the 230 K Au films exhibited increased membrane disruption and higher levels of photothermal-induced cellular damage following visible-light illumination. Conversely, the Au films fabricated at 300 K demonstrated comparatively weaker cytotoxic effects, consistent with their reduced plasmonic behavior observed in the optical characterization experiments. Quantitative analysis of PI-positive cells relative to total DAPI-stained nuclei is presented in Figure 3E. The descriptive quantitative analysis showed a higher proportion of PI-positive cells for the cryogenically fabricated Au nanoparticle films compared with the conventionally fabricated films. The enhanced biological response observed for the 230 K samples is attributed to the highly uniform nanoparticle organization and strong plasmonic coupling generated through the proposed soliton-mediated nonequilibrium growth mechanism. These observations suggest that the improved structural homogeneity and plasmonic behavior associated with cryogenic nonequilibrium growth may contribute to the more pronounced photothermal response observed for the 230 K Au nanoparticle films.

Since the most pronounced photothermal cytotoxic response was observed on Au nanoparticle-coated surfaces fabricated at 230 K, subsequent experiments focused on comparing the responses of cancerous A549 cells and noncancerous BEAS-2B cells under identical irradiation conditions. The simultaneous evaluation of A549 cells and noncancerous BEAS-2B epithelial cells was particularly important for assessing the preferential response under the investigated experimental conditions for the plasmonic photothermal platform and determining whether the cryogenically fabricated Au nanoparticle films could induce preferential photothermal damage toward cancer cells while minimizing nonspecific cytotoxicity in normal epithelial cells. Figure 4A,D schematically illustrate the proposed photothermal responses of A549 and BEAS-2B cells cultured on cryogenically fabricated Au nanoparticle-coated substrates under visible-light illumination. Cells were seeded at a density of 5 × 10^4^ cells per substrate and incubated overnight at 37 °C under a humidified 5% CO2 atm. Samples were subsequently exposed to 600 nm light for 10 min using a xenon light source adjusted to the plasmon resonance wavelength of Au nanoparticles (13–15 nm). Images were acquired under identical imaging conditions using a 20× objective with 80% digital zoom. Upon excitation within the plasmonic resonance region, localized photothermal heating generated by the Au nanoparticle assemblies induces membrane stress and cellular destabilization. Due to enhanced plasmonic coupling and localized heat confinement, the photothermal effect is expected to be substantially more pronounced in cancer cells than in noncancerous epithelial cells.

**Figure 4:**
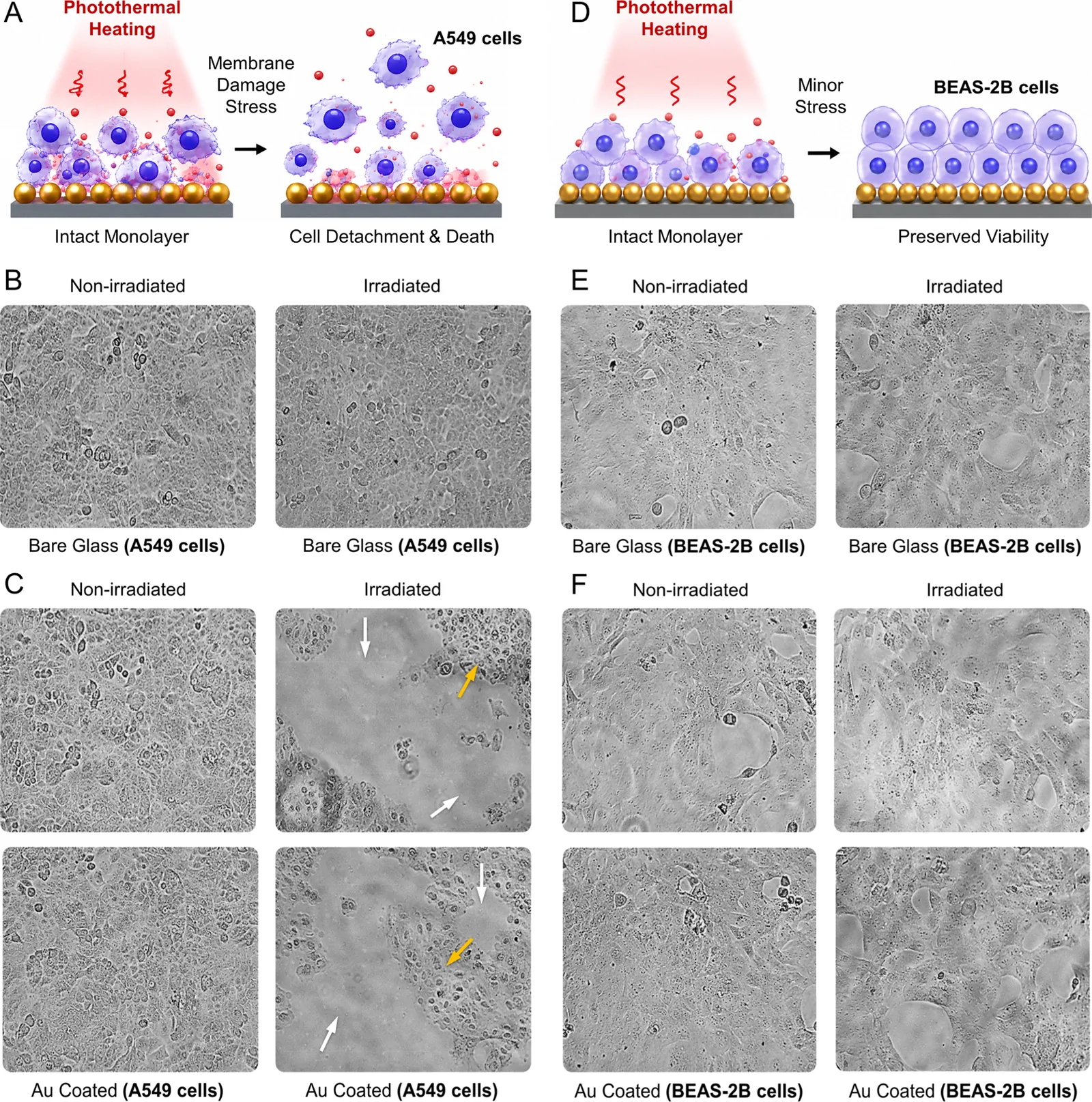
(A,D) Schematic illustrations of the proposed photothermal response of A549 and BEAS-2B cells on Au nanoparticle-coated surfaces under plasmon-mediated heating conditions. Representative brightfield microscopy images of A549 and BEAS-2B cells cultured on bare-glass (B,E) and Au nanoparticle-coated glass substrates (C,F) under nonilluminated and illuminated conditions, respectively.

As shown in Figure 4B,C, A549 cells exhibited pronounced morphological disruption following illumination on Au nanoparticle-coated substrates fabricated at 230 K. In contrast, nonirradiated control samples maintained dense and homogeneous cellular organization. Brightfield microscopy of irradiated A549 cells revealed the formation of extensive cell-depleted regions and localized loss of surface adhesion on Au-coated substrates. Distinct cell-free zones were observed after illumination (white arrows), accompanied by disruption of intercellular junctions and deterioration of cohesive monolayer organization at the boundaries of the damaged regions (yellow arrows). Importantly, the observed cytotoxic response was not strictly confined to the directly illuminated regions, suggesting lateral thermal propagation and plasmon-mediated local heat diffusion across neighboring cellular areas. Furthermore, A549 cells cultured on Au nanoparticle-coated substrates displayed pronounced morphological abnormalities after irradiation, including reduced cell spreading, irregular cellular morphology, decreased intercellular connectivity, and partial collapse of the confluent cellular architecture. These observations indicate that localized plasmonic heating generated by the cryogenically fabricated Au nanoparticle films induced substantial membrane stress and structural destabilization within the cancer-cell population.

In contrast, BEAS-2B cells exhibited comparatively limited morphological alterations under identical photothermal treatment conditions, as shown in Figure 4E,F. Both nonirradiated and irradiated BEAS-2B cells largely preserved their cellular attachment, surface coverage, and monolayer organization on both bare-glass and Au-coated substrates. Only minor local morphological disturbances were observed following illumination, indicating substantially lower photothermal susceptibility compared with A549 cancer cells. The simultaneous evaluation of cancerous A549 cells and noncancerous BEAS-2B cells was particularly important for assessing the preferential response under the investigated experimental conditions for the cryogenic plasmonic platform, demonstrating that the fabricated Au nanoparticle films preferentially induced photothermal damage in cancer cells while largely preserving normal epithelial-cell integrity under identical irradiation conditions. Importantly, the enhanced photothermal response observed in A549 cells on cryogenically fabricated Au nanoparticle-coated surfaces further supports the role of nanoparticle uniformity, dense packing, and strong localized electromagnetic coupling in promoting efficient plasmon-mediated heat generation. Compared with bare-glass controls, Au nanoparticle-coated substrates produced a more pronounced biological response to 600 nm light exposure in A549 cells, whereas BEAS-2B cells exhibited comparatively limited alterations under the same experimental conditions. These findings suggest that the observed preferential response of A549 cells may arise from the combined effects of enhanced plasmonic heating and intrinsic biological differences between cancerous and noncancerous cells, including their adhesion behavior, membrane properties, and thermal stress responses.

Fluorescence-based membrane integrity analysis further revealed a more pronounced photothermal response in A549 cells than in BEAS-2B cells under the investigated experimental conditions. As schematically illustrated in Figure 5A,B, visible-light illumination of Au nanoparticle-coated surfaces induced markedly different biological responses in A549 and BEAS-2B cells. A549 cells exhibited extensive membrane damage, cellular detachment, and reduced recovery during the postirradiation period, whereas BEAS-2B cells showed comparatively minor stress responses and preserved cellular viability following recovery. Representative brightfield and fluorescence microscopy images are presented in Figure 5C,D. Fluorescence-based membrane integrity analysis revealed extensive membrane damage in A549 cells immediately after illumination, as demonstrated by intense PI-positive staining at Day 0 (Figure 5C). Brightfield imaging additionally showed reduced cellular attachment and morphological disruption following photothermal treatment. Although the proportion of PI-positive cells slightly decreased by Day 3, substantial membrane damage remained detectable, indicating persistent photothermal injury within the surviving cell population. This reduction in PI-positive staining may result from the loss and detachment of severely damaged cells together with partial recovery of the remaining viable cells over time.

**Figure 5:**
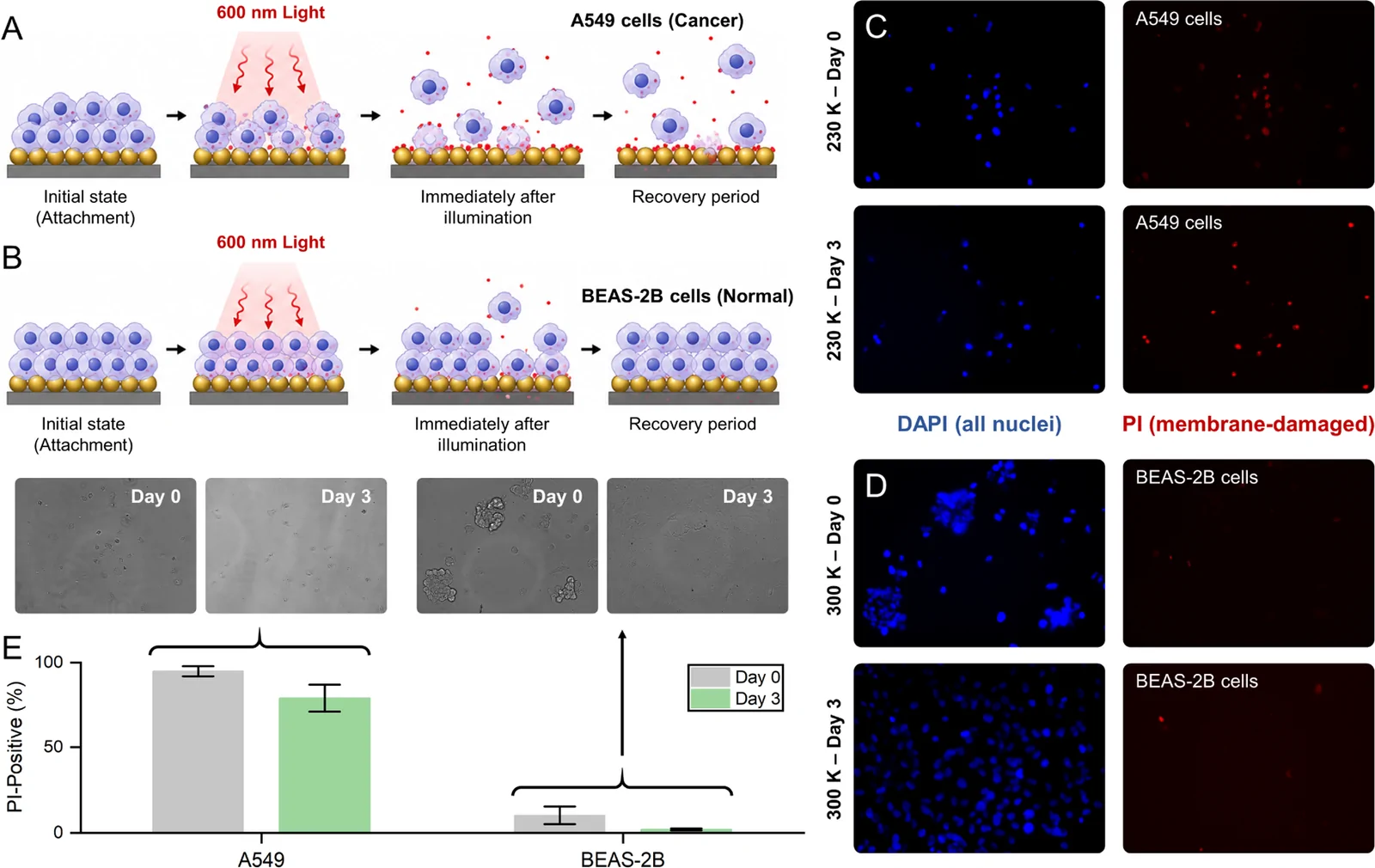
(A,B) Schematic illustrations of the proposed photothermal responses of A549 cancer cells and BEAS-2B normal epithelial cells, respectively, under 600 nm light irradiation. (C,D) Representative DAPI-stained (blue), and PI-stained (red) fluorescence images of detached A549 and BEAS-2B cells acquired immediately after illumination (day 0) and after the recovery period (day 3), respectively. (E) Quantitative analysis of PI-positive cells normalized to total DAPI-stained nuclei for A549 and BEAS-2B cells. Data are presented descriptively as mean ± SD, and no inferential statistical analysis was performed. Figure inset shows the brightfield images of A549 and BEAS-2B cells on Au nanoparticle-coated substrates.

In contrast, BEAS-2B cells exhibited minimal PI-positive staining at both Day 0 and Day 3, indicating preservation of membrane integrity and limited photothermal-induced cell death (Figure 5D). Furthermore, brightfield imaging demonstrated that BEAS-2B cells were capable of reattaching and proliferating during the recovery period following illumination, suggesting substantially greater resistance to plasmon-mediated photothermal stress compared with A549 cancer cells. Quantitative analysis of PI-positive cells normalized to total DAPI-stained nuclei is presented in Figure 5E. A549 cells exhibited extremely high levels of membrane damage immediately after treatment, with approximately 95% PI-positive cells at Day 0 and persistent membrane damage (∼79%) remaining detectable at Day 3. In contrast, BEAS-2B cells displayed minimal membrane damage, with only ∼10% PI-positive cells at Day 0 and ∼2% at Day 3. These descriptive results indicate a more pronounced photothermal response in A549 cells than in BEAS-2B cells under the investigated experimental conditions. The parallel evaluation of A549 and BEAS-2B cells therefore provides preliminary evidence of different cellular responses to the cryogenically fabricated plasmonic platform under the investigated conditions.

Overall, Au nanoparticle-coated surfaces enhanced the cellular response to 600 nm illumination, with A549 cells exhibiting more pronounced membrane disruption, cell detachment, and photothermal-induced cellular damage than BEAS-2B cells under the investigated conditions. This differential response may be influenced by both the plasmonic properties of the Au nanoparticle films and intrinsic biological differences between the two cell lines. These preliminary observations support further investigation of cryogenically fabricated Au nanoparticle films as plasmonic photothermal platforms using expanded biological models and increased experimental replication.

The enhanced susceptibility of A549 cells to plasmon-mediated photothermal treatment compared with BEAS-2B cells is likely multifactorial. Previous studies have shown that cancer cells generally exhibit altered cytoskeletal organization, reduced mechanical stiffness, increased membrane fluidity, and higher metabolic activity compared with their normal counterparts, making them more vulnerable to thermal stress.^53^ In addition, differences in cell morphology and adhesion behavior may influence the extent of contact between the cells and the plasmonic Au nanoparticle surface, thereby affecting local heat transfer during irradiation.^54^ A549 cells typically display a larger spreading area and more intimate interaction with the substrate than BEAS-2B cells, which may enhance exposure to localized plasmonic heating. Furthermore, cancer cells often possess a reduced capacity to recover from heat-induced membrane damage and oxidative stress, whereas normal epithelial cells generally exhibit more effective repair mechanisms that facilitate membrane restoration and reattachment following transient thermal injury.^55^ Therefore, the differential photothermal responses observed between A549 and BEAS-2B cells in the present study are likely influenced by the combined effects of differences in cell–substrate interactions, membrane properties, cytoskeletal organization, and intrinsic thermal tolerance rather than by a single biological factor. Although the present work was not designed to investigate these molecular mechanisms directly, the observed behavior is consistent with previous reports describing the greater sensitivity of cancer cells to localized photothermal stress.^19,56^

To directly evaluate the photothermal heating generated during irradiation, temperature changes were monitored using a Fluke Ti32 infrared camera during photothermal treatment performed at room temperature (300 K). For the cell-free bare-glass, 300 K Au/glass, and 230 K Au/glass substrates, the temperature changes during irradiation were below the sensitivity of the infrared camera and therefore could not be reliably quantified. In contrast, under the cell-based photothermal experimental conditions, a measurable temperature increase of approximately 5–7 °C was detected, reaching a plateau within approximately 100 s. The temperature increased rapidly during the initial irradiation period, reaching its maximum within approximately 100 s, after which it remained relatively stable. This rapid temperature increase and subsequent plateau demonstrate light-induced heat generation by the Au nanoparticle-coated surface and provide direct experimental support for the proposed plasmon-mediated photothermal mechanism. The observed saturation behavior may reflect the establishment of a steady-state balance between photothermal heat generation and heat dissipation to the surrounding environment. Although A549 cells exhibited substantially greater photothermal-induced membrane damage than BEAS-2B cells under identical experimental conditions, the present study was designed to compare the responses of one cancerous and one noncancerous epithelial cell line rather than to establish universal cancer selectivity. The observed differences are likely influenced by multiple biological factors, including variations in cell adhesion, membrane properties, cytoskeletal organization, thermal stress sensitivity, and recovery capacity. Therefore, while the present results demonstrate a preferential photothermal response of A549 cells under the investigated conditions, further studies involving additional cancer and normal cell models will be required to determine the broader applicability of this behavior.

## 4 Discussion

In this study, a cryogenic nonequilibrium deposition strategy was developed for the fabrication of plasmonic Au nanoparticle films with enhanced photothermal therapeutic performance. The novelty of the proposed approach lies in the utilization of cryogenic substrate cooling during Au nanoparticle growth, enabling the formation of highly uniform plasmonic nanostructures under nonequilibrium deposition conditions. FESEM analysis performed over a broad substrate-temperature range (100–300 K) showed that deposition at 230 K produced densely packed and highly uniform Au nanoparticle assemblies. These morphological characteristics, together with the AFM and XRD observations, are consistent with the proposed soliton-mediated nonequilibrium growth model; however, they do not constitute direct experimental proof of this mechanism. Under these cryogenic nonequilibrium conditions, the resulting Au films consisted of densely packed and nearly uniform metallic nanoparticle assemblies, leading to enhanced structural homogeneity and enhanced plasmonic coupling behavior. Optical characterization demonstrated that the cryogenically fabricated Au nanoparticle films exhibited a pronounced SPR response centered near 600 nm, which was attributed to the highly uniform nanoparticle organization obtained under conditions consistent with the proposed soliton-mediated growth regime. In contrast, Au films fabricated under conventional growth conditions displayed broader particle-size distributions and comparatively weaker plasmonic behavior due to heterogeneous island-type nanoparticle formation.

The photothermal therapeutic performance of the fabricated plasmonic films was systematically evaluated using both A549 lung cancer cells and BEAS-2B normal bronchial epithelial cells cultured on bare-glass, 300 K Au-coated, and 230 K Au-coated substrates. The simultaneous investigation of cancerous and noncancerous cell lines was particularly important for evaluating the preferential photothermal response of the plasmonic platform under identical experimental conditions. Photothermal treatment was performed under visible-light illumination at 600 nm using a 450 mW xenon light source for 10 min. Morphological analysis, fluorescence imaging, and membrane-integrity assays suggested that illumination of cryogenically fabricated Au nanoparticle-coated substrates induced substantial cytotoxic effects in A549 cancer cells, including localized cell loss, disruption of cell–cell interactions, membrane damage, and increased PI-positive staining. In contrast, BEAS-2B cells remained comparatively resistant under identical treatment conditions and largely preserved their membrane integrity, cellular organization, and recovery capability during the postirradiation period.

The enhanced photothermal response observed for the cryogenically fabricated Au nanoparticle films is likely associated with strong plasmonic coupling and localized electromagnetic hot-spot generation arising from the highly uniform nanoparticle arrangement obtained under cryogenic deposition conditions. Importantly, the different responses observed between A549 and BEAS-2B cells indicate that, under the investigated experimental conditions, the cryogenically fabricated plasmonic platform produced a greater photothermal response in A549 cells than in BEAS-2B cells, rather than indiscriminate cytotoxicity. These observations demonstrate the potential of cryogenically fabricated plasmonic Au nanoparticle films for further investigation as photothermal platforms in future in vitro and preclinical studies. Overall, this study demonstrates that cryogenic nonequilibrium deposition produces Au nanoparticle films with improved structural homogeneity and enhanced plasmonic behavior. Under the investigated in vitro conditions, these films exhibited a preferential photothermal response in A549 cells relative to BEAS-2B cells, while preserving comparatively higher viability of the normal epithelial cells. The present biological evaluation should be regarded as a proof-of-concept investigation. Although the observed trends were consistent across the performed experiments, additional biological replicates and expanded statistical analyses will be valuable in future studies to further validate the observed preferential photothermal response. Our findings demonstrate a preferential photothermal response of A549 cells relative to BEAS-2B cells under the investigated experimental conditions. Nevertheless, additional studies involving multiple cancer and normal cell models, direct thermal measurements, expanded biological replication, and mechanistic investigations will be necessary to establish the generality and underlying biological basis of this preferential response and to further evaluate its potential for photothermal biomedical applications.

## Abbreviations

AFM: atomic force microscopy
Au: gold
A549: human lung adenocarcinoma cells
BEAS-2B: normal bronchial epithelial cells
CO_2_: carbon dioxide
DAPI: 4′,6-diamidino-2-phenylindole
EGFR: epidermal growth factor receptor
FBS: fetal bovine serum
FESEM: field-emission scanning electron microscopy
NSCLC: nonsmall cell lung carcinoma
PBS: phosphate-buffered saline
PI: propidium iodide
PTT: photothermal therapy
R: reflectance
Ra: average surface roughness
Rq: root-mean-square roughness
SCLC: small cell lung carcinoma
SPR: surface plasmon resonance
T: transmittance
